# Inorganic Trace Analysis by Isotope Dilution Mass Spectrometry—New Frontiers

**DOI:** 10.6028/jres.093.100

**Published:** 1988-06-01

**Authors:** J. D. Fassett

**Affiliations:** Center for Analytical Chemistry, National Bureau of Standards, Gaithersburg, MD 20899

Isotope dilution mass spectrometry (IDMS) is used extensively at NBS in the certification of elemental concentrations in Standard Reference Materials. It is regarded at NBS as a “definitive method,” that is, a method of proven high accuracy. Since the theme of this symposium is accuracy in trace element analysis, it is appropriate to review the role IDMS plays in accurate inorganic analysis. The thesis of this paper is that mass spectrometry is a dynamic technique, marked by continuous productive activity and change, and that new mass spectrometric methods and new ionization techniques promise to make IDMS more general, more available, and more cost effective. Furthermore, I argue that the adoption of this technique by analytical laboratories outside of reference laboratories would do much to broaden the accuracy base of the world’s measurements.

In IDMS the quantity of an element present in a material is determined from the change produced in the isotopic composition of the element when a known amount of stable isotope (called a spike) is added. Thus, the technique is applicable to all elements with more than one stable isotope, or greater than 60 elements in the periodic table. In practice this number is reduced to those elements readily handled and ionized in the source of a mass spectrometer. In addition, some mononuclidic elements can be determined using radioactive isotopic spikes. For instance, procedures have been developed in our laboratory for iodine and thorium using ^129^I and ^230^Th.

An IDMS requires equilibrium of the spike isotope and the natural isotopes, and thus requires that the sample be dissolved. It is at this stage of the procedure that the potential sources of systematic error are most likely to occur. Clearly, if the sample does not completely dissolve, or, if the spike or sample isotopes are lost before equilibration, or, if contamination occurs in the dissolution process, the measured isotopic ratio will not reflect the accurate ratio of added spike atoms/sample atoms for that element. The analyst must be aware of these pitfalls.

The analyte concentration is determined knowing the isotopic composition of the spike and sample, the concentration of the spike, the weights of the mixed spike and sample, and the measured ratio. Thus, the spike must be calibrated. This calibration is accomplished doing a reverse IDMS procedure using gravimetrically prepared natural solutions from high purity materials. Also, both the natural and isotopic compositions must be verified.

The major source of separated isotopes is Oak Ridge National Laboratory [1], although there are alternative sources for some isotopes. The Electromagnetic Isotope Enrichment Facility (EMIEF) at Oak Ridge is currently used to produce 225 enriched stable isotopes of 50 elements. The isotopes in the sales inventory are for sale to anyone on a first-come, first-served basis. The cost of a separated isotope is roughly proportional to the extent of enrichment. However, the cost of the isotopic spike in IDMS is a minor part of the analysis and paradoxically becomes even less so in trace and ultratrace measurements. For instance, the ^50^V spike costs $131/mg. The cost of the *μ*g of this spike used in spiking a sample with V at the ppm level is only 13 cents. More important, perhaps, is the future cost and availability of enriched stable isotopes. This issue has recently been discussed [2].

IDMS has traditionally been done using: thermal ionization mass spectrometry (TIMS) [3], an instrumental method used primarily in the nuclear and geochemical fields; spark source mass spectrometry (SSMS) [3], primarily at NBS; and, GC/MS electron impact ionization [4,5], using more widely available organic mass spectrometry instrumentation and techniques. There have been important advances in the traditional techniques and commercial instrumentation that are valuable in accurate, trace measurement using IDMS including: new procedures, higher sensitivity, and higher precision.

Examples of new thermal ionization procedures for IDMS are ones for sulfur [6] and vanadium [7]. The sulfur technique is novel in that the AsS^+^ ion is generated and measured. The sulfur isotopes are shifted 75 mass units higher in the mass spectrum by the mononuclidic arsenic moiety. This technique has resulted in over 50 certification measurements since its development, illustrating the previous void in analytical techniques that could accurately measure sulfur at the parts-per-million level. The vanadium method uses pulse-counting TIMS and has been used to certify various materials from the ppb level to minor levels. This procedure illustrates one of the advantages of high-sensitivity IDMS procedures: large dynamic range capability. The sample is optimally spiked according to the concentration of the element of interest. Then, a subsample of the separated element is often adequate for determination of the altered isotopic ratio.

An illustration of the sensitivity achievable using TIMS is illustrated in the procedures developed for uranium [8]. The concentration of uranium in bovine liver (SRM 1577a) has been determined at the 700 pg/g level with a few percent precision from equivalent 0.25 g sample sizes. For these high sensitivity measurements, it is clear that accurate measurements require control and characterization of the analytical blank. Kelly has presented this topic at this symposium. The use of well-characterized, sub-boiling-distilled reagents helps keep reagent blanks to a minimum. The development of efficient and specific chemical separations, often involving microchemical procedures, has further helped to lower the limit of measurement for many elements.

Commercial instrumentation in TIMS now possess multicollectors and multisample turrets, as well as full automation. The multicollectors can demonstrate parts in 10^6^ internal precision in favorable cases. Although this precision may be wasted in IDMS analysis, the traditional requirement for the production of long-lived, stable ion beams to reach high precision is considerably reduced. In many cases, this advance could reduce the need for very high purity elemental separations. Thus, the advances in commercial instrumentation promise to increase the throughput, reduce the labor costs, and, in general, reduce the overall cost of an IDMS measurement. In addition, one commercial vendor is selling a quadrupole-based thermal ionization instrument. Although not capable of the extremely high precision possible using a magnetic sector TIMS instrument, this relatively low cost instrument can make isotope ratio measurements with nominal 0.25% precision, certainly very adequate in many instances for accurate IDMS.

The general advances that have been made in sample pretreatment, automation, and separations that have been discussed at this symposium promise to reduce the front end labor costs of IDMS. More importantly, these advances could also do much to reduce the contamination that occurs in routine chemical operations. The very high sensitivity of many of the new and traditional mass spectrometric techniques will only be applicable when the limits imposed by the blank are overcome.

There have been a number of presentations at this symposium where the “new frontiers” of IDMS can be seen. Recently, two new techniques have been examined using isotope dilution: resonance ionization mass spectrometry (RIMS) and inductively coupled plasma mass spectrometry (ICP-MS). RIMS uses laser photoionization to produce ions from a gas phase reservoir of atoms [9]. Both the sensitivity and elemental selectivity of RIMS can be used to advantage in isotope ratio measurement. ICP-MS combines high sensitivity with breadth of elemental coverage, and, direct analysis of nebulized solutions [10]. The speed and convenience of the ICP-MS, and elemental coverage, in combination with its rapid commercialization give the analytical community an unprecedented opportunity to apply IDMS, and achieve accurate trace element analysis.
